# Multi-Source Cooperative Data Collection with a Mobile Sink for the Wireless Sensor Network

**DOI:** 10.3390/s17112493

**Published:** 2017-10-30

**Authors:** Changcai Han, Jinsheng Yang

**Affiliations:** School of Microelectronics, Tianjin University, Tianjin 300072, China; cchan@tju.edu.cn

**Keywords:** wireless sensor networks, mobile sink node, cooperative communications, sparse cooperation, distributed low-density parity-check codes

## Abstract

The multi-source cooperation integrating distributed low-density parity-check codes is investigated to jointly collect data from multiple sensor nodes to the mobile sink in the wireless sensor network. The one-round and two-round cooperative data collection schemes are proposed according to the moving trajectories of the sink node. Specifically, two sparse cooperation models are firstly formed based on geographical locations of sensor source nodes, the impairment of inter-node wireless channels and moving trajectories of the mobile sink. Then, distributed low-density parity-check codes are devised to match the directed graphs and cooperation matrices related with the cooperation models. In the proposed schemes, each source node has quite low complexity attributed to the sparse cooperation and the distributed processing. Simulation results reveal that the proposed cooperative data collection schemes obtain significant bit error rate performance and the two-round cooperation exhibits better performance compared with the one-round scheme. The performance can be further improved when more source nodes participate in the sparse cooperation. For the two-round data collection schemes, the performance is evaluated for the wireless sensor networks with different moving trajectories and the variant data sizes.

## 1. Introduction

For traditional wireless sensor networks (WSNs), many static nodes are randomly deployed and the data of active sensor nodes are usually forwarded to the sink node through multiple hops [1]. However, this transmission strategy brings a series of problems. On the one hand, the energy consumption of different sensor nodes is not uniform and more energy may be consumed for the nodes closer to the sink node compared with those located farther away from the sink due to the converge-cast traffic pattern [2,3,4,5]. In this way, the network lifetime may degrade and thus the connectivity or the coverage of wireless sensor networks can not be guaranteed [2]. On the other hand, the data rate of the multi-hop transmission decreases as the number of hops increases and it is also a challenging topic to maximize the throughput for some WSNs [6,7].

Mobile sink nodes were introduced to overcome some challenges above for wireless sensor networks [4,5,6,7,8,9]. In this way, the energy consumption of different sensor nodes is balanced and thus the network lifetime can be prolonged [5,8,9]. The throughput can also be maximized by employing mobile sinks in some wireless sensor networks [7]. For mobile sink networks, the energy consumption can be further reduced by designing efficient wireless communication strategies because the data sending and receiving by communication modules may consume the major energy in WSNs [10]. Therefore, the power-efficient data transmission schemes should be investigated to further prolong the lifetime of wireless sensor networks with mobile sinks. For example, the virtual multiple-input multiple-output was employed to gather mobile data in [11]. Cooperative diversity with network coding was used to improve the communication reliability in [12]. In this paper, we devise the data collection schemes with low power consumption by using cooperative communication technologies such as multi-source cooperation and coded cooperation for wireless sensor networks with mobile sinks. The schemes are based on the cooperative effort of sensor nodes, which is recognized as an important feature of wireless sensor networks [1].

Cooperative communications can achieve the diversity gain to decrease the impairment of wireless fading channels and improve the power efficiency by sharing antennas of different nodes in wireless networks [13,14]. In cooperative networks, different relay protocols such as amplify-and-forward (AF), decode-and-forward (DF) and coded cooperation (CC) have been designed for cooperative nodes to forward the received data to the destination. For coded cooperation, it can achieve the diversity gain and the coding gain simultaneously by integrating traditional coding schemes into cooperative communications [15]. Various coded cooperation schemes have been designed based on different coding schemes such as low-density parity-check (LDPC) codes, turbo codes, generalized low-density codes and network codes [16,17,18,19,20,21,22,23,24,25]. For example, turbo codes were employed in wireless relay networks to design distributed coding schemes [16]. Low-density parity-check codes were investigated for relay channels in [17] and generalized low-density codes were used in multi-relay networks [18]. Generalized adaptive network coded cooperation strategy based on LDPC codes was proposed for cooperative networks [21] and network coding was used to implement efficient multi-source cooperation schemes in [22]. For performance evaluation, we have implemented a distributed testbed with software-defined radios to test coded cooperation schemes in a real indoor wireless environment [23].

The investigations above disclose that coded cooperation can achieve significant performance gain for wireless networks. For multi-node coded cooperation, each node usually needs multiple cooperative partners so as to obtain the good performance. However, it is difficult for the complex cooperation relation to be implemented for wireless sensor networks due to the limited energy and processing capability of sensor nodes. Therefore, efficient multi-node coded cooperation schemes with low complexity should be elaborately designed for wireless sensor networks to reduce the energy consumption and improve the lifetime.

In this paper, multi-source sparse cooperation schemes with efficient distributed LDPC codes are proposed to transmit data from multiple sources to the mobile sink in WSN. We investigate two cooperation models. In the first model, each source node usually has one parter to help itself and it also assists another partner, where the moving sink traverses one time along the distribution region of the active source nodes. In the second model, each source node is helped by two partners and also participates in the data transmission of two cooperative partners, where the moving sink traverses twice in the region of source nodes. We call the two cooperation models as multi-source sparse cooperation due to the simple cooperation relation especially when a lot of source nodes participate in the cooperation. Specifically, the sparse cooperation models are firstly formed considering the geographical locations of the nodes, the quality of the inter-node wireless channels, and the moving trajectories of the mobile sink node. Here, the neighbors of source nodes can be chosen to serve as the cooperative partners. Then, directed graphs and cooperative matrices are involved to present the cooperation models. Finally, distributed LDPC codes based on the sparse cooperation are devised to jointly transmit the data from multiple sources to the mobile sink node. In this cooperative data collection schemes, the energy consumption of the sensor nodes can be greatly reduced and each source node has very low complexity. If more source nodes participate in the sparse cooperation, the performance can be further improved.

This paper is organized as follows. Section 2 introduces two multi-source cooperation models according the moving trajectories of the mobile sink. In Section 3, the cooperation models are firstly presented by directed graphs and cooperative matrices, and then distributed quasi-cyclic LDPC codes are constructed to match the sparse cooperation models. In Section 4, the performance of the proposed schemes are evaluated by simulations. Finally, some conclusions are drawn in Section 5.

## 2. Multi-Source Sparse Cooperation Models with a Mobile Sink

**Figure 1:**
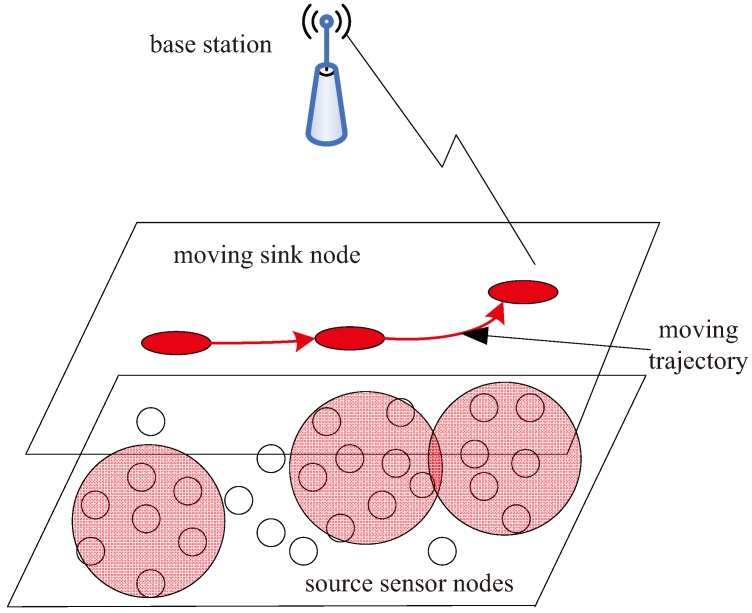
Architecture of a wireless sensor network with a mobile sink.

We consider a wireless sensor network consisting of a large quantity of static sensor nodes and a mobile sink node connecting to the remote base station, as shown in Figure 1. The sink node moves around the region where the sensor nodes are located to collect the data in the wheel-moving or jumping mode [26,27]. We devise the multi-source coded cooperation schemes to improve the power efficiency for wireless sensor networks.

The network model is defined as follows. In a wireless sensor network, multiple sensor nodes $s_{1},s_{2},\ldots ,s_{N}$ need to transmit their data to a common mobile sink node *d* moving along the distribution region of source nodes. Assume that each source node $s_{i},1\leq i\leq N$ is constrained to the half-duplex mode and transmits signals on the orthogonal channel such as time division multiple access (TDMA) or code division multiple access (CDMA). We assume TDMA is used for different source nodes in this paper. Each inter-source channel denoted by $s_{i}-s_{j},i\neq j$ and each source-sink channel represented by $s_{i}-d$ are modeled as independent quasi-static Rayleigh fading channels. For these channels, the fading factors stay constant during a frame and vary from one frame to another.

For the wireless sensor network above, we investigate cooperative models, where *N* sensor nodes consist a cooperative set $S=\left\{s_{1},s_{2},\ldots ,s_{N}\right\}$ to jointly transmit data to the mobile sink node *d*. In this paper, we design two sparse cooperative models according to different moving trajectories of the sink node. In the first model, the moving sink node *d* moves along the distribution region of the active source nodes one time only. For the other model, the mobile sink *d* moves twice in the distribution region of the nodes along different routes. Indeed, the two moving trajectories interlace with one another. How to form the cooperative models are explained in the following.

### 2.1. One-Round Cooperation Model

**Figure 2:**
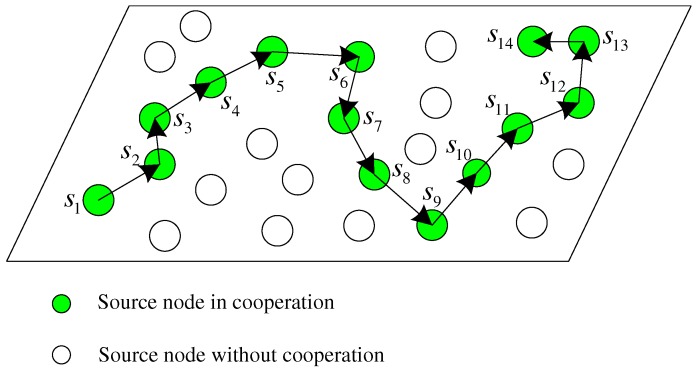
Multi-source sparse cooperation model with one-round moving of the sink node.

In this model, each source node $s_{i},1\leq i\leq N$ broadcasts test data to the sink node *d* and its neighbors sequentially when the mobile sink *d* approaches and awakes it. Specifically, when the source node $s_{i}$ transmits signals, its neighbors and the sink node *d* listen to $s_{i}$. Let $R(s_{i})$ denote the node set, in which the member nodes can correctly receive the data from the source $s_{i}$. It is assumed that at least one neighbor can obtain the correct data from the node $s_{i}$ and this assumption is not difficult to be guaranteed in densely-deployed wireless sensor networks. If multiple neighbor nodes successfully receive the data from the node $s_{i}$, we choose only one node $s_{j}\in R\left(s_{i}\right)$ as the partner of the node $s_{i}$ among all the candidates. The selected node $s_{j}$ volunteers to not only assist the node $s_{i}$ but also transmit its own data to the approaching sink node *d* during the data collection. In this way, a cooperative link is formed one by one as shown in Figure 2. Note that the cooperation model should be formed based on the moving trajectory of the sink node *d* and the reception results $R(s_{i})$, 1≤i≤N, for some nodes may fail to decode the data of some other sources due to the fading and noise of inter-source channels.

In Figure 2, it is a line-like cooperation model, where each source node in the cooperative link except the first node $s_{1}$ and the last node $s_{N}$ has a single parter to help itself and it also assists only another partner. For the two special nodes, the first node $s_{1}$ assisted by the node $s_{2}$ does not help any node, while the last node $s_{N}$ participating the data transmission of the node $s_{N-1}$ has no node to assist itself. Without loss of generality, the description about the cooperation relation refers to the general source nodes in this paper. We assume that such a cooperative link can be formed in the densely-deployed wireless sensor networks and the specific partner selection strategies are beyond the scope of the paper. Indeed, the cooperative network can be formed based on the geographical information of the source nodes, the inter-node channels, and the moving trajectory of the sink node. In Figure 2, the filled nodes denote the active nodes in this line-like cooperative model and the unfilled nodes denote the sleeping nodes in this snapshot. In this paper, we assume that the source nodes with data to upload are all included in the cooperative link and the sleeping nodes do not have data to transmit at this moment. Indeed, if some source nodes not included in the active link have data to report, they can transmit data to the nearest active nodes to assist them uploading the data through the active cooperative link. This is out of the investigation of this paper and we only aim at the joint data collection from the active nodes in the cooperative link.

For the cooperative link, we will design effective coded cooperation scheme with LDPC codes to achieve significant performance with quite low complexity in the next section. Note that the cooperative link in Figure 2 is quite different from the traditional multi-hop transmission. On the one hand, each node $s_{i}$ in the link jointly encodes and transmits its own data and the correctly received data from its selected partner during the cooperation, while the relay usually simply forwards the partner data in the traditional multi-hop transmission. On the other hand, the data rate does not decrease as *N* increases and the performance can be improved when more source nodes participate in the cooperative link in Figure 2. However, the transmission rate in the multi-hop transmission reduces as the number of hops increases.

### 2.2. Two-Round Cooperation Model

**Figure 3:**
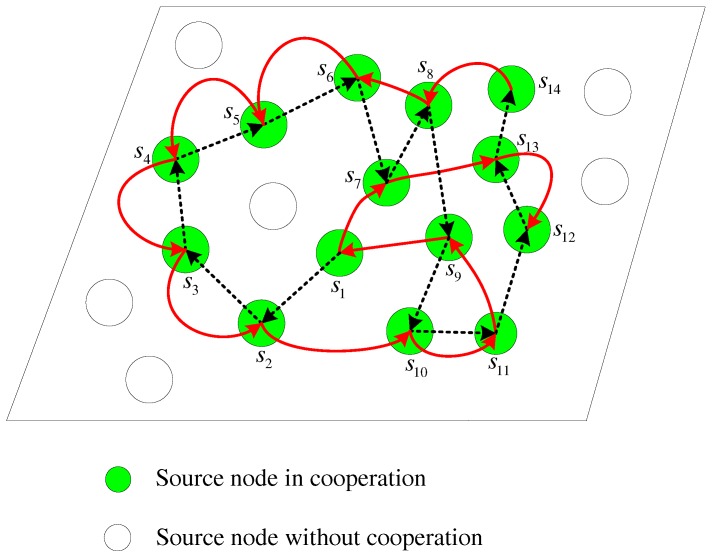
Multi-source sparse cooperation model with two-round moving of the sink node.

In this model, the moving sink node traverses twice along the distribution region of the active source nodes $s_{1},s_{2},\ldots ,s_{N}$. The two rounds are along the different trajectories and construct different cooperative links. Each cooperative link can be similarly formed as in the one-round cooperation model above. Here, each source node also performs in sequence when the mobile sink node approaches and awakes it. Figure 3 illustrates an example with 14 active sensor nodes. In the figure, the dashed and solid lines depict the two different cooperative links during the two rounds, respectively. It is shown that each node except three special nodes including the first node $s_{1}$ in the first round, the last nodes $s_{14}$ in the first round and the last node $s_{12}$ in the second round, has two parters to help itself and also assists two partners. For the node $s_{j}\in S$, we assume it assists $s_{i}$ and $s_{k}$ in the two rounds, respectively. For example, the node $s_{6}$ assists $s_{5}$ and $s_{8}$ in the first round and the second round, respectively, as illustrated in Figure 3. For the special node $s_{1}$, it is assisted by $s_{2}$ and $s_{7}$ and only helps $s_{9}$. For the special nodes $s_{14}$ and $s_{12}$, the node $s_{14}$ only has one parter $s_{8}$ to assist it and helps another node $s_{13}$, while the node $s_{12}$ helps two parters $s_{11}$ and $s_{13}$ and only has the parter $s_{13}$ to assist itself.

In order to obtain the promising performance, the second trajectory of the moving sink node *d* should be different from the first one, though the two trajectories both traverse the nodes one by one in the cooperative group $S=\left\{s_{1},s_{2},\ldots ,s_{N}\right\}$. Moreover, when multiple neighbors can successfully obtain the data from the broadcasting source nodes, the parter selection algorithm in each step during the two rounds may affect the overall performance. In this paper, we mainly address the strategy of cooperative data collection and the optimal parter selection algorithm will not be investigated.

Indeed, each moving round plays different roles in the cooperative data collection. In the next section, an efficient distributed encoding scheme is designed using LDPC codes to further illustrate the two-round cooperation model. Generally, for the first moving round, the sink node collects the information data broadcasted by each source node sequentially and each source node keeps the correctly received information data from its partner according to the dashed line in Figure 3. For the second round, each source node receives the encoded parity-check bits from its partner according to the solid line in Figure 3, and then jointly encodes its own data and the data collected from its selected partners during the two rounds. For the sink node, it collects the parity-check bits transmitted by each source node sequentially during the second round. The cooperative processing and encoding procedure will be explained in detail in the following section.

## 3. Directed Graphs and Distributed Low-Density Parity-Check Codes for Data Collection

In this section, distributed low-density parity-check (LDPC) codes are designed based on the multi-source sparse cooperation models above to achieve the diversity gain and coding gain simultaneously. Specifically, the node cooperation models are firstly described using directed graphs. Then, the coded cooperation schemes with LDPC codes are devised based on the directed graphs. In the design, the coding schemes are constructed with the expansion from the basic matrix, which can be derived from the cooperative models. In this way, the data of all the sources in the cooperative group $S=\left\{s_{1},s_{2},\ldots ,s_{N}\right\}$ are encoded in a fully distributed and concise way and jointly transmitted to the mobile sink.

### 3.1. Directed Graphs for Multi-Source Sparse Cooperation

In the following, we represent the cooperation relation using the directed graphs for the two models.

#### 3.1.1. One-Round Cooperation Graph

**Figure 4:**
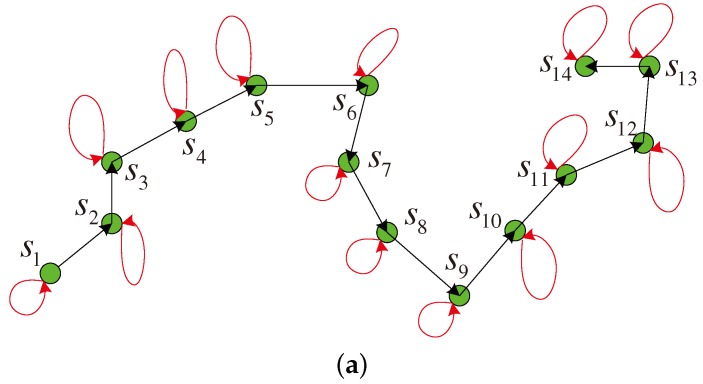

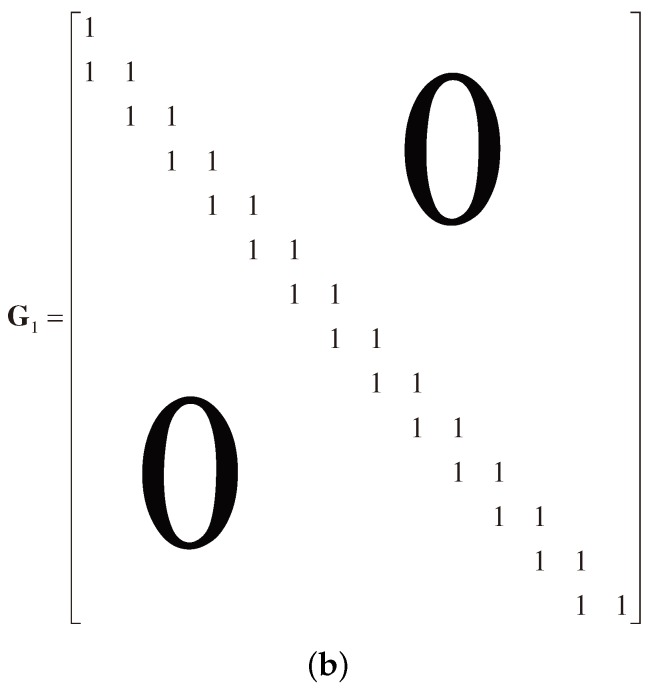
Directed graph and cooperation matrix for the one-round cooperation model (**a**) directed graph; (**b**) cooperation matrix.

In this line-like cooperation model, each source node except $s_{1}$ and $s_{N}$ is aided by one source node and also assists another node. In this paper, we propose a directed graph to represent the cooperation relation. Figure 4a illustrates the directed graph for the cooperative model in Figure 2. If there is a directed edge $e_{ij}=\left(s_{i},s_{j}\right)$ in the graph, it means that the source node $s_{j}$ can successfully receive the data from $s_{i}$ and the node $s_{j}$ volunteers to jointly encode and forward the data from $s_{i}$ and its own data. In Figure 4a, there is a loop for each node $s_{i},1\leq i\leq N$, for each node participates in the transmission of its own data. Therefore, we can devise the cooperation topology on a directed graph D=(S,E), where the set *S* consists of all the nodes in the cooperative link and the edge set *E* defines the cooperative relation among all the nodes.

Then, let define a N×N cooperation matrix $G_{1}$ given by

(1) $$G_{1}\left(D\right)={\left[g_{j,i}^{1}\right]}_{N\times N},$$

where *N* is the number of the nodes involved in the cooperation, and $g_{j,i}^{1}=1$ denotes a directed edge from $s_{i}$ to $s_{j}$. If there is a directed edge from $s_{i}$ to $s_{j}$, the source $s_{j}$ helps the source $s_{i}$ forward the data to the destination. There is a directed edge from the source $s_{i}$ to $s_{i}$, i.e., the element $g_{i,i}^{1}=1$, which means that $s_{i}$ participates its own data transmission. Figure 4b illustrates the cooperation matrix $G_{1}$ for the directed graph in Figure 4a. It is obvious that the bidiagonal matrix $G_{1}$ is a sparse matrix for the large *N* in the one-round cooperation model. Thus, we call it the multi-source sparse cooperation in this paper. In the following, the matrix $G_{1}$ is used to design the basic matrix for the distributed LDPC code.

#### 3.1.2. Two-Round Cooperation Graph

**Figure 5:**
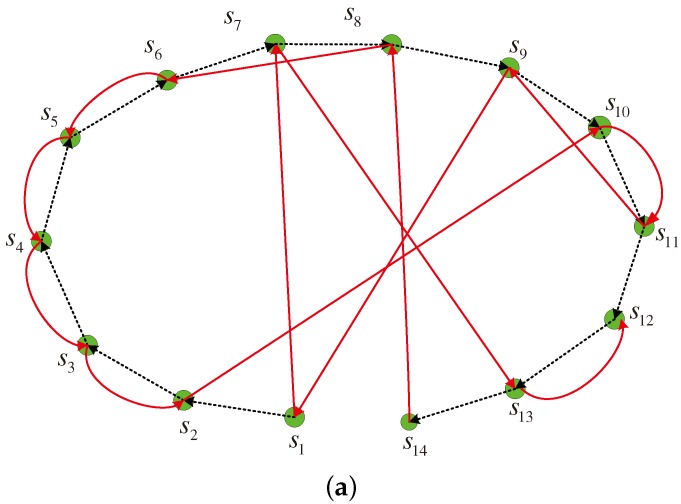

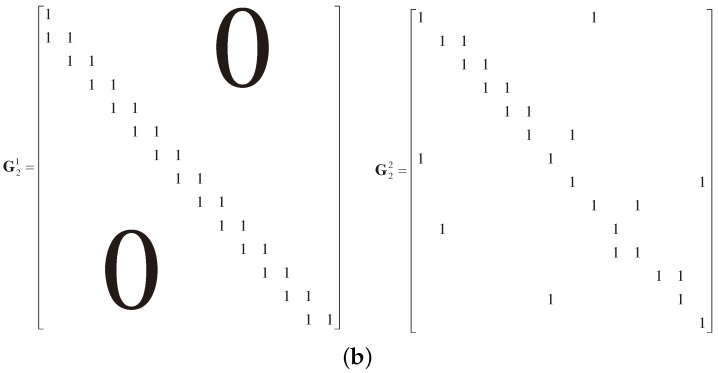
Directed graph and cooperation matrices for the two-round cooperation model (**a**) directed graph; (**b**) cooperation matrices for two rounds.

In this model, the moving sink node traverses twice along the distribution region of the active source nodes. In this way, the source nodes cooperate during the two rounds and the cooperation relation can be also denoted using the directed graphs. Taking the two-round cooperation model in Figure 3 as an example, the directed graph and the cooperation matrix are illustrated in Figure 5. It is noted that there are two self-loops eliminated at each source node for conciseness.

For the directed graph in Figure 5a, define the matrix $G_{2}^{1}={\left[g_{j,i}^{2,1}\right]}_{N\times N}$ and the matrix $G_{2}^{2}={\left[g_{j,i}^{2,2}\right]}_{N\times N}$ to denote the cooperation relation in the first round and the second round, respectively, as illustrated in Figure 5b. Here, the element $g_{j,i}^{2,1}=1$ in the matrix $G_{2}^{1}$ and $g_{j,i}^{2,2}=1$ in $G_{2}^{2}$ denote the directed edges from the node $s_{i}$ to the node $s_{j}$ for the directed graph in Figure 5a. The element $g_{i,i}^{2,1}=1$ and $g_{i,i}^{2,2}=1$ mean that $s_{i}$ participates its own data transmission in the both rounds, though the self-loops are omitted in Figure 5a for conciseness. In the following, let the overall cooperation matrix $G_{2}=\left[G_{2}^{1},\;G_{2}^{2}\right]$ as the basic matrix and efficient distributed LDPC codes can be designed in the following.

### 3.2. Cooperative Data Collection with Distributed LDPC Codes

When multi-source cooperation graphs are formed, low complex distributed processing schemes using distributed LDPC codes are proposed to match the directed graphs *D*. Overall, each source node in the set *S* jointly encodes its own data and the data successfully received from one or two selected partners according to the cooperation graphs and then forwards the encoded bits to the mobile sink. The detailed procedures for two cooperation models are introduced as follows.

#### 3.2.1. Distributed LDPC Codes for One-Round Collection

For the cooperative data collection with the one-round model, each source node in the cooperative link acts sequentially when the sink node *d* approaches it. Generally, each source node only receives the information data from its prior node according to the cooperation graph *D* and combines the data with its own data to generate the parity check data. For the line-like cooperative data collection, the processing at source nodes consists of two phases.

In the first phase, each source node $s_{i}$ broadcasts its own information data frame $m_{i}$ of *K* bits to its selected partner $s_{j}\in R\left(s_{i}\right)$ according to the directed graph *D* and the mobile sink node *d*, when the sink approaches and awakes $s_{i}$. The information data $m_{i}$ may be encoded using a block code or directly transmitted without protection according to the inter-source channel quality. Without loss of generality, it is assumed that no channel coding is used during this phase in this paper.

In the second phase, the selected partner $s_{j}\in R\left(s_{i}\right)$ combines its own data $m_{j}$ with the correctly decoded data $m_{i}$ from the node $s_{i}$ to generate the parity-check data vector $p_{j}$. Specifically, the vector $p_{j}$ is calculated by

(2) $$p_{j}^{T}=A^{-1}\left(\Pi_{j,i}m_{i}^{T}+\Pi_{j,j}m_{j}^{T}\right),$$

where $\Pi_{j,i}$ denotes a K×K randomly-permuted identity matrix and *A* is a K×K bidiagonal matrix given by

(3) $$A=\left[\begin{array}{ccccc} 1 & & & & \\ 1 & 1 & & & \\ & 1 & 1 & & \\ & & & \ddots & \\ & & & 1 & 1 \end{array}\right].$$

The linear time encoding can be implemented at each source node in a distributed manner. The parity-check data vector $p_{j},1\leq j\leq N$ is transmitted by the parter $s_{j}\in R\left(s_{i}\right)$ to the mobile sink node *d*. Thus, the parity-check data vectors $p_{1},p_{2},\ldots ,p_{N}$ can be generated and forwarded sequentially by all the cooperative source nodes $s_{1},s_{2},\ldots ,s_{N}$ in a distributed and sequential manner according to the cooperation graph *D*.

In this way, the mobile sink node *d* collects the data transmitted from all the sources in the set $S=\{s_{1},s_{2},\ldots ,s_{N}\}$ to form a whole codeword denoted as $c=\left[m,p\right]$, where $m=\left[m_{1},m_{2},\ldots ,m_{N}\right]$ and $p=\left[p_{1},p_{2},\ldots ,p_{N}\right]$. Let us analyze the codeword c received at the destination. Define a matrix $h_{j}=\left[h_{j,1},h_{j,2},\ldots ,h_{j,N}\right]$, where $h_{j,i}=\Pi_{j,i}$ if $g_{j,i}^{1}=1$ in the cooperation matrix $G_{1}$ as illustrated in Figure 4b and otherwise $h_{j,i}=0_{K\times K}$. Thus, Equation (2) is transformed into

(4) $$p_{j}^{T}=A^{-1}h_{j}m^{T}.$$

Define a sparse matrix

(5) $$h=\left[\left.\begin{array}{c} h_{1}\\ h_{2}\\ \vdots \\ h_{N} \end{array}\right|\begin{array}{cccc} A & & & \\ & A & & \\ & & \ddots & \\ & & & A \end{array}\right],$$

satisfying

(6) $${Hc}^{T}=h{\left[m,p\right]}^{T}=0^{T}.$$

Therefore, the whole codeword c received at the mobile sink *d* from *N* cooperative sources during two phases is indeed a codeword of the LDPC code with *h* as the parity-check matrix and the matrix *h* can be easily derived from the cooperation matrix $G_{1}$ according to Equation (5). Here, the code rate is 1/2 which stays constant for the variant *N* of cooperation graphs. Then, the iterative decoding can be performed at the sink node *d* to decode the distributed LDPC code and obtain the data of all the sources in *S*. Moreover, the encoding and transmission are implemented by all the sources in the cooperative group $S=\left\{s_{1},s_{2},\ldots ,s_{N}\right\}$ in a distributed manner and thus the complexity of each source node is low. Thus, the line-like cooperative data collection in Figure 4 is quite different from the traditional multi-hop data transmission. In addition, if channel codes are also used at *N* sources to protect the information data vector *m* during the first phase, they can be combined with the distributed LDPC code to further improve the performance.

#### 3.2.2. Distributed LDPC Codes for Two-Round Collection

In this model, the sink node *d* moves two rounds along the region of active sensor nodes in the set *S* and two different cooperative links of two rounds also play different roles in the data collection. During each round, each source node also acts sequentially when the sink node *d* approaches it according to its related cooperative link in the graph *D*. The processing at the source nodes during the two rounds is described as follows, which is different from the one-round model above.

In the first moving round, each source node $s_{i}$ just broadcasts its own information data frame $m_{i}$ with *K* bits to the mobile sink node *d* and the selected partner in the first round according to the directed graph *D*. Each node just keeps the correctly decoded data from its partner and does not generate the parity check bits during the first round. For example, we assume that the node $s_{j}\in S$ assists $s_{i}$ during the first round, and thus the node $s_{j}$ just stores the information data $m_{i}$ received from the node $s_{i}$ for the further encoding in next round.

In the second moving round, each node generates and forwards the parity check bits using its own information data, the information data from its partner in the first round, and the parity-check data from its another partner in the second round. Taking the node $s_{j}$ as an example, we assume that it assists $s_{i}$ and $s_{k}$ in the first round and the second round, respectively. The parity-check vector $p_{j}$ at the node $s_{j}$ can be jointly generated by its own information data $m_{j}$, the information data $m_{i}$ from $s_{i}$ in the first round, and the parity-check data $p_{k}$ from $s_{k}$ in the second round. Thus, the vector $p_{j}$ can be given by

(7) $$p_{j}^{T}=\left(\Pi_{j,i}^{1}m_{i}^{T}+\Pi_{j,j}^{1}m_{j}^{T}\right)+\Pi_{j,k}^{2}p_{k}^{T},$$

where $\Pi_{j,i}^{1}$, $\Pi_{j,j}^{1}$, and $\Pi_{j,k}^{2}$ are K×K randomly-permuted identity matrices. Then, the parity check vector $p_{j}$ can be transmitted to the mobile sink *d* by the node $s_{j}$. Here, the generation and transmission of the parity check vectors $p_{1},p_{2},\ldots ,p_{N}$ are performed sequentially by the source nodes $s_{1},s_{2},\ldots ,s_{N}$ in a distributed and sequential manner. Indeed, for the encoding at each source node, we only need to sum all the permuted bits according to Equation (7). Therefore, the encoding complexity at each node is quite low. However, the disadvantage is the long storage time requirement including its own source data and the data from the partners during the two rounds.

In this way, the sink node *d* combines the information data $m=\left[m_{1},m_{2},\ldots ,m_{N}\right]$ collected in the first round and the parity check data $p=\left[p_{1},p_{2},\ldots ,p_{N}\right]$ in the second round to form a whole codeword $c=\left[m,p\right]$, which is also a LDPC codeword. The iterative decoding can be performed for the sink node. Here, the code rate is r=1/2, which is the same as the one-round model. The parity-check matrix *h* of the whole code can easily be obtained from the cooperative matrices $G_{2}^{1}$ and $G_{2}^{2}$ in Figure 5. In the following, we will simulate the performance of distributed LDPC codes with the expansion of the randomly-permuted identity matrix.

## 4. Performance Evaluation with Simulations

In this section, the performance of the proposed multi-source cooperative data collection schemes with the mobile sink node is evaluated by simulation. We assume each active source node can successfully receive the data from at least two neighbor source nodes and the sparse cooperation models mentioned above can be formed for the wireless networks with uniform random deployment of dense sensor nodes. In the simulation, Binary Phase Shift Keying (BPSK) constellations are employed and the data from each source node undergo independent and identically distributed quasi-static Rayleigh fading. We compare the performance of the one-round cooperation, the two-round cooperation, and the non-cooperation data collection scheme. In the non-cooperation data collection scheme, each source node individually transmits its data to the sink node by employing an LDPC code with the same code code length and code rate with the cooperative schemes, when the sink node approaches and awakes it. The iterative decoding based on message passing with maximum iteration of 30 times is performed at the sink node *d* for all the data collection schemes. The simulation stopping criterion is that the maximum number of frame is 1,000,000 or the error frame number reaches 100 for all the simulations.

**Figure 6:**
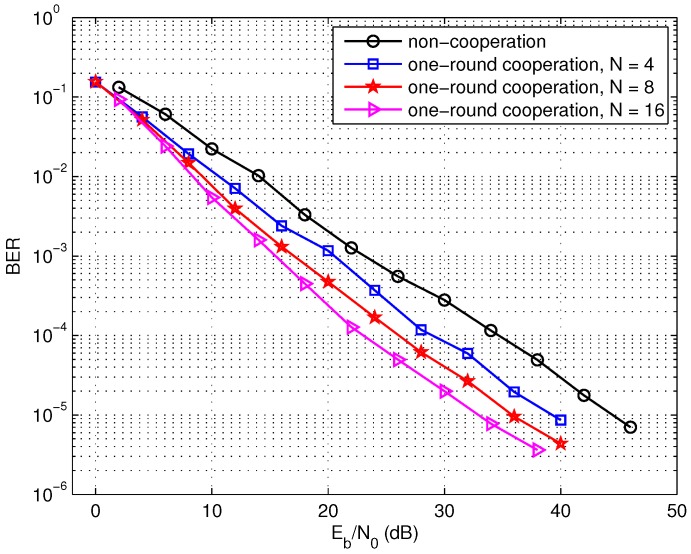
Performance of the one-round sparse cooperation with different number of source nodes, L=1024 bits.

Firstly, the bit error rate (BER) of the one-round cooperation scheme is compared with the non-cooperation scheme in Figure 6. The overall code length *L* in all the schemes equals to 1024 bits and the code rate is r=1/2. When the number *N* of the source nodes involved in the cooperation set *S* increases, the data length *K* of each source decreases with the constraint L=K×N/r in order to maintain the constant code length *L* for comparison. We simulate three one-round cooperation cases with different *N*, that is, the cooperation with 4, 8, or 16 nodes. In Figure 6, it is observed that the one-round sparse cooperation with distributed LDPC codes exhibits better performance over the non-cooperation scheme. It can be also observed that the performance of the cooperation scheme improves with the increasing of the cooperative node number *N*. Moreover, the encoding complexity at each source node in the one-round sparse cooperation scheme is lower than the non-cooperation, for distributed encoding is employed in the cooperation scheme.

**Figure 7:**
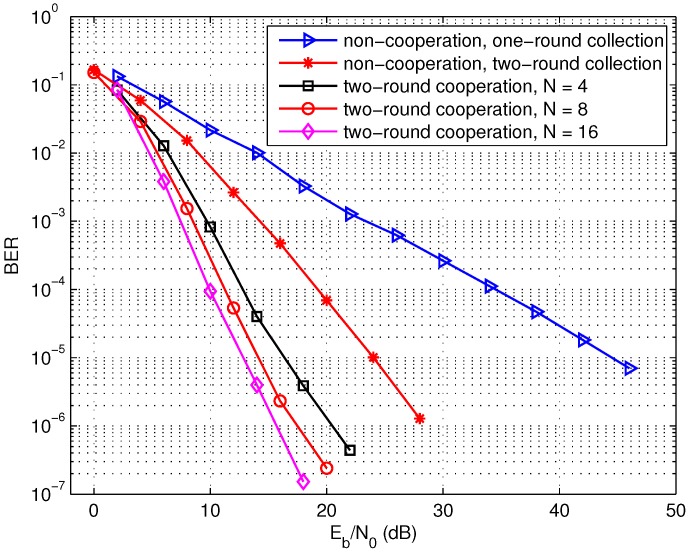
Performance of the two-round sparse cooperation with different number of source nodes, L=1024 bits.

Secondly, the BER performance of the two-round cooperation with different numbers of source nodes is illustrated in Figure 7. Here, we assume the source-sink channels denoted by $s_{i}-d,1\leq i\leq N$ keep constant during the same moving round of the sink node and change for two rounds due to the different locations of the sink node. It is observed that the two-round cooperation with four sources can achieve about 28 dB gain over the non-cooperation scheme with one round moving of the sink node at a BER of $10^{-5}$. It can also further improve the performance by employing more cooperative source nodes. For the non-cooperation, the sink node can also move two rounds to collect data and the non-cooperation with two-round moving improves the performance over one-round scheme, attributed to the diversity in Figure 7. In addition, the proposed cooperative data collection schemes lead to some degree of complexity such as the routing protocol compared with the non-cooperation scheme.

**Figure 8:**
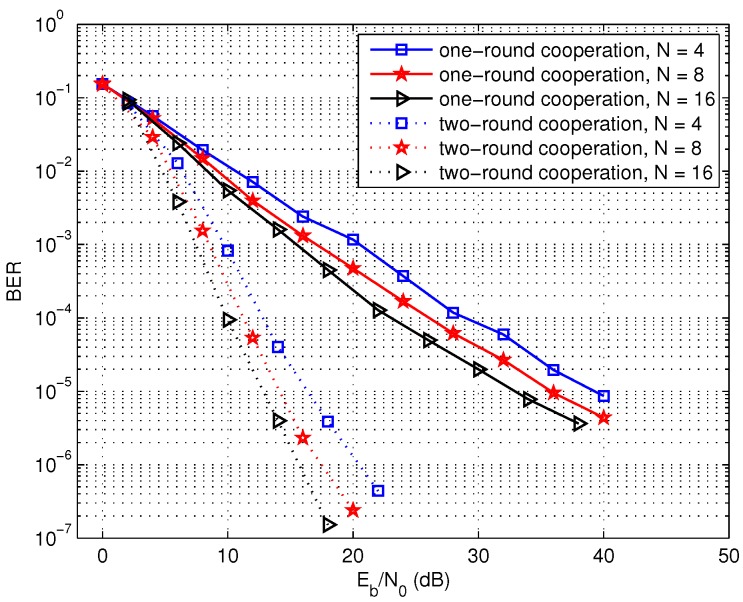
Performance comparison of the one-round cooperation and the two-round cooperation schemes, L=1024 bits.

Thirdly, the performance of the two multi-source cooperation models is compared in Figure 8. It can be observed that the two-round cooperative scheme can achieve much better performance over the one-round cooperation with the same number of cooperation nodes. For example, the two-round cooperation scheme with eight source nodes can achieve about 21 dB gain over the one-round scheme. The performance improvement is attributed to two reasons. On the one hand, additional diversity gain can be obtained due to the wireless channel change when the sink node moves twice along the region of source node. One the other hand, a little more complex cooperation relation is formed so as to construct more efficient distributed LDPC codes when the sink node approaches each source node twice in wireless sensor networks.

**Figure 9:**
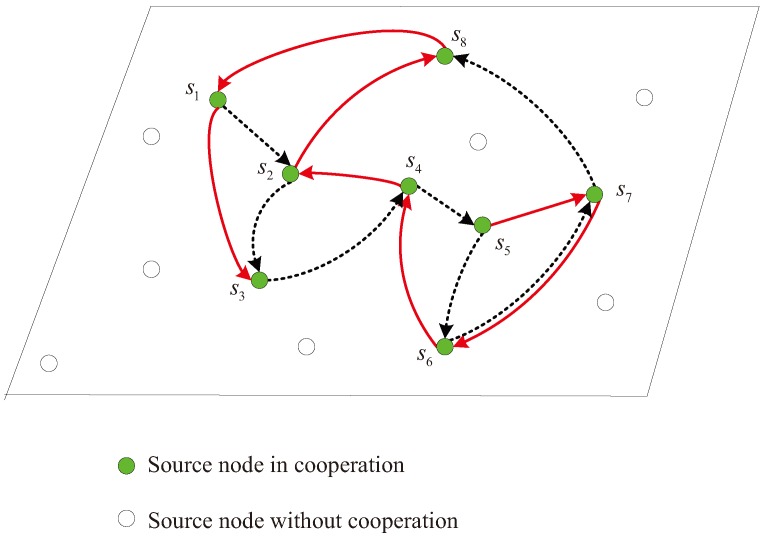
Moving trajectory I (solid lines) of the mobile sink during the second round, *N* = 8.

**Figure 10:**
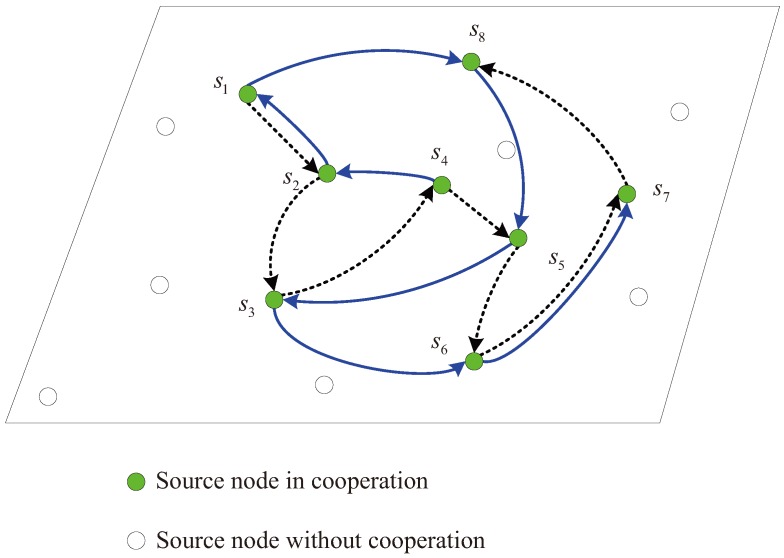
Moving trajectory II (solid lines) of the mobile sink during the second round, *N* = 8.

**Figure 11:**
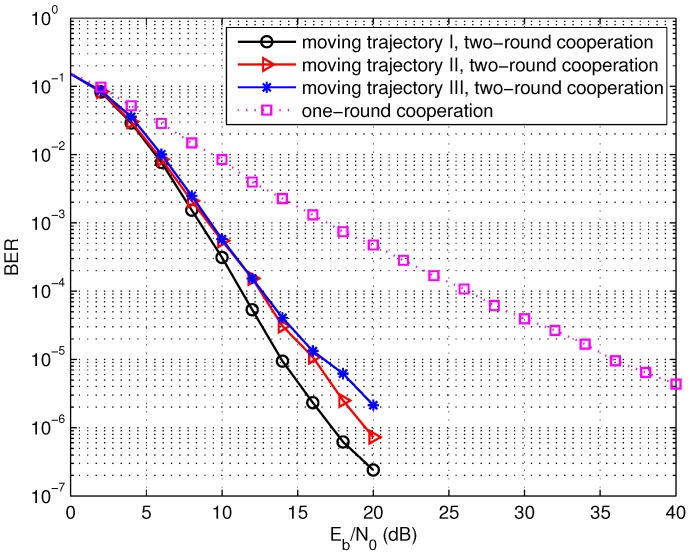
Performance of the the two-round cooperation schemes with different moving trajectories of the mobile sink, *N* = 8, L=1024 bits.

Fourthly, the moving trajectories of the sink node are discussed. We have addressed that the moving trajectory during the second round should be different from the trajectory in the first round so as to construct the distributed LDPC code with good performance. Taking the network with N=8 cooperative nodes as an example, the different moving trajectories I and II for the second round are illustrated in Figure 9 and Figure 10, respectively. Here, the moving trajectories for the second round are denoted by the solid lines and the trajectories for the first round denoted by the dashed lines are the same in the both figures. Figure 11 shows the BER performance of the two-round cooperation schemes with different moving trajectories of the sink node. It is observed that the moving trajectory I achieves a little better performance than the trajectory II. There is one small cycle consisting of $S_{6}$ and $S_{7}$ in Figure 9, while the nodes $S_{1}$, $S_{2}$, $S_{6}$ and $S_{7}$ compose two small cycles of the distributed LDPC code for the moving trajectory II in Figure 10. It is well known that the small cycles decrease the performance of the LDPC codes. The small cycles may decrease the BER performance of the distributed LDPC codes, even though the influence is reduced, attributed to the extension for the parity-check matrix. Therefore, small cycles should be formed as few as possible by optimizing the moving trajectory of the sink node and the cooperative partner selection of each source node. In addition, such small cycles can be greatly reduced as the number of cooperative nodes increases.

**Figure 12:**
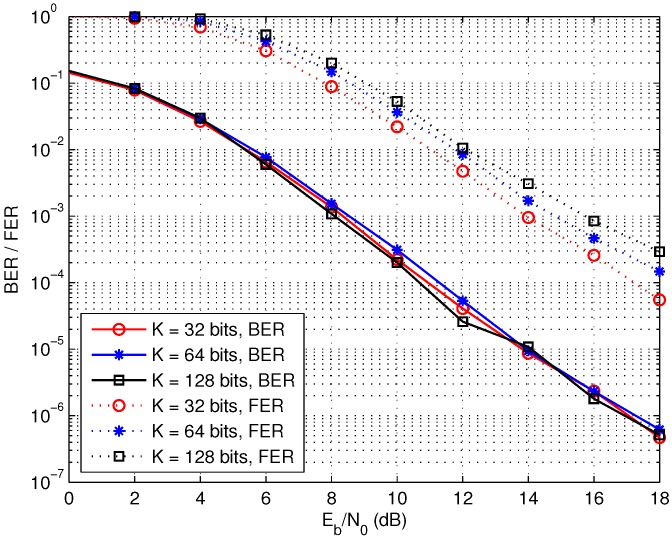
Performance of the two-round cooperation schemes with different data length, *N* = 8.

Finally, the performance of the two-round cooperation schemes with different data sizes is compared in Figure 12. On the one hand, the increasing of the information data length *K* results in the longer LDPC codes with the code length L=K×N/r at the sink node, which improves the BER performance attributed the coding gain. On the other hand, we assume the data from each source node undergo independent and identically distributed quasi-static Rayleigh fading, which varies from one frame to another. Thus, the block fading channels also vary when the frame length changes. Specifically, the increasing of the frame length also enlarges the fading block, which decreases the BER performance of the distributed LDPC codes. Here, the BER performance is almost the same for the three different data sizes because the improved coding gains may be used to compensate for the worse block fading channels. The frame error rate (FER) performance is also given in Figure 12. It is observed that the FER performance decreases as the frame length increases. Indeed, the influence of the data size on the performance is complicated because it is difficult to evaluate the performance of the LDPC codes under block fading channels with the variant block sizes. In addition, the time-delay also increases for the bigger frame and thus the data size should be chosen considering these issues in practical wireless sensor networks.

## 5. Conclusions

For the large scale wireless sensor networks with one mobile sink node, multi-source sparse cooperation schemes integrating distributed LDPC codes are proposed to jointly collect different source data to the sink node. Specifically, two cooperation models are formed considering the geographical locations of the source nodes, practical channel condition and the moving trajectories of the sink node. Then, directed graphs and cooperation matrices are derived for the one-round and two-round cooperation models and the distributed LDPC codes are designed to match different cooperation models. Finally, simulation results are given to exhibit the significant performance of the proposed one-round and two-round cooperation schemes compared with the non-cooperation, while each source node has very low processing complexity attributed to the distributed encoding and transmission in the cooperation schemes. Furthermore, the performance of the two-round data collection schemes is also evaluated considering different moving trajectories and the variant data sizes.
